# Gain and loss of function mutations of ataxin-7 cause cilia pathology in mouse and zebrafish models

**DOI:** 10.1186/2046-2530-4-S1-P13

**Published:** 2015-07-13

**Authors:** A Karam, R Ferreira, C Weber, L Morlé, J Vermot, Y Trottier

**Affiliations:** 1 Translational Medicine and Neurogenetics, IGBMC, Illkirch, France; 2 Development and Stem Cells, IGBMC, Illkirch, France; 3 Centre de Génétique et de Physiologie Moléculaires et Cellulaires, Lyon, France

## 

Spinocerebellar ataxia type 7 (SCA7) is an autosomal dominant neurodegenerative disease characterized by progressive neuronal loss in the cerebellum and associated structures, leading to cerebellar ataxia, dysarthria and dysphagia. Additional non-cerebellar symptoms are present at variable frequencies, including visual impairment affecting 83% of patients that is caused by cone and rod photoreceptor degeneration and hearing loss concerning 24% of patients.

SCA7 is caused by a toxic polyglutamine expansion in Ataxin-7, a known subunit of the transcriptional co-regulator SAGA/TFTC complex. Transcriptional alterations are observed in SCA7, however, the disease mechanism is yet unclear. Ataxin-7 is also present in the cytoplasm of neurons, where its function is unknown.

Here, we show for the first time that Ataxin-7 is a bona fide component of centrosomes and is present in diverse types of mammalian cilia, including primary, motile and photoreceptor sensory cilia. We provide evidence that Ataxin-7 loss of function causes a large spectrum of ciliary-related morphological and functional phenotypes in zebrafish, and that human Ataxin-7 is able to rescue the cilia phenotypes, demonstrating that Ataxin-7 has an essential, evolutionarily conserved function in cilia. Strikingly, Ataxin-7 is progressively lost from photoreceptor cilia in SCA7 mouse models, correlating with ciliary defects and photoreceptor degeneration. Finally, we found that expression of polyQ-expanded Ataxin-7, like Ataxin-7 loss of function, leads to ciliary dysfunctions in zebrafish. Our study thus provides new insight into the function of Ataxin-7 and opens novel perspective in the understanding of SCA7 pathogenesis.

